# Supplementary material for: Bayesian Group Index Regression for Modeling Chemical Mixtures and Cancer Risk
Source: Int J Environ Res Public Health. 2021 Mar 27;18(7):3486. doi: 10.3390/ijerph18073486 (PMC8037139; doi:10.3390/ijerph18073486)
Supplement: Supplementary file 1 [file ijerph-18-03486-s001.pdf]

Bayesian Group Index Regression for Modeling Chemical Mixtures and Cancer Risk

David C. Wheeler[a], Salem Rustom[a], Matthew Carli[a], Todd Whitehead[b], Mary H. Ward[c], Catherine Metayer[b]

[a]Department of Biostatistics, School of Medicine, Virginia Commonwealth University, Richmond, VA

[b]UC Berkeley School of Public Health, Berkeley, CA

[c]Occupational and Environmental Epidemiology Branch, Division of Cancer Epidemiology and Genetics, National Cancer Institute, Rockville, MD

Table S1: Individual scenarios in the scenarios sets of the simulation study

		No. chemicals in each group			No. important in each group			Association strength, OR				
No.	Set	Group 1	Group 2	Group 3	Group 1	Group 2	Group 3	Group 1	Group 2	Group 3	Sample size, N	Correlation structure, r
1	A	5	4	0	2	2	0	1.00	1.00	-	1000	0.1 in group, 0.5 across groups
2	A	5	4	0	2	2	0	0.67	1.50	-	1000	0.1 in group, 0.5 across groups
3	A	5	4	0	2	2	0	0.50	2.00	-	1000	0.1 in group, 0.5 across groups
4	A	5	4	0	2	2	0	0.40	2.50	-	1000	0.1 in group, 0.5 across groups
5	A	5	4	0	2	2	0	0.33	3.00	-	1000	0.1 in group, 0.5 across groups
6	A	5	4	0	2	2	0	1.00	1.00	-	1000	0.3 in group, 0.7 across groups
7	A	5	4	0	2	2	0	0.67	1.50	-	1000	0.3 in group, 0.7 across groups
8	A	5	4	0	2	2	0	0.50	2.00	-	1000	0.3 in group, 0.7 across groups
9	A	5	4	0	2	2	0	0.40	2.50	-	1000	0.3 in group, 0.7 across groups
10	A	5	4	0	2	2	0	0.33	3.00	-	1000	0.3 in group, 0.7 across groups
11	A	5	4	0	2	2	0	1.00	1.00	-	1000	0.5 in group, 0.9 across groups
12	A	5	4	0	2	2	0	0.67	1.50	-	1000	0.5 in group, 0.9 across groups
13	A	5	4	0	2	2	0	0.50	2.00	-	1000	0.5 in group, 0.9 across groups
14	A	5	4	0	2	2	0	0.40	2.50	-	1000	0.5 in group, 0.9 across groups
15	A	5	4	0	2	2	0	0.33	3.00	-	1000	0.5 in group, 0.9 across groups
16	B	5	4	5	1	1	1	1.00	1.00	1.00	1000	0.1 in group, 0.5 across groups
17	B	5	4	5	1	1	1	0.67	1.50	1.50	1000	0.1 in group, 0.5 across groups
18	B	5	4	5	1	1	1	0.50	2.00	2.00	1000	0.1 in group, 0.5 across groups
19	B	5	4	5	1	1	1	0.40	2.50	2.50	1000	0.1 in group, 0.5 across groups
20	B	5	4	5	1	1	1	0.33	3.00	3.00	1000	0.1 in group, 0.5 across groups
21	B	5	4	5	1	1	1	1.00	1.00	1.00	1000	0.3 in group, 0.7 across groups
22	B	5	4	5	1	1	1	0.67	1.50	1.50	1000	0.3 in group, 0.7 across groups
23	B	5	4	5	1	1	1	0.50	2.00	2.00	1000	0.3 in group, 0.7 across groups
24	B	5	4	5	1	1	1	0.40	2.50	2.50	1000	0.3 in group, 0.7 across groups
25	B	5	4	5	1	1	1	0.33	3.00	3.00	1000	0.3 in group, 0.7 across groups
26	B	5	4	5	1	1	1	1.00	1.00	1.00	1000	0.5 in group, 0.9 across groups
27	B	5	4	5	1	1	1	0.67	1.50	1.50	1000	0.5 in group, 0.9 across groups
28	B	5	4	5	1	1	1	0.50	2.00	2.00	1000	0.5 in group, 0.9 across groups
29	B	5	4	5	1	1	1	0.40	2.50	2.50	1000	0.5 in group, 0.9 across groups
30	B	5	4	5	1	1	1	0.33	3.00	3.00	1000	0.5 in group, 0.9 across groups
31	C	5	4	5	3	2	3	1.00	1.00	1.00	1000	0.1 in group, 0.5 across groups
32	C	5	4	5	3	2	3	0.67	1.50	1.50	1000	0.1 in group, 0.5 across groups
33	C	5	4	5	3	2	3	0.50	2.00	2.00	1000	0.1 in group, 0.5 across groups
34	C	5	4	5	3	2	3	0.40	2.50	2.50	1000	0.1 in group, 0.5 across groups
35	C	5	4	5	3	2	3	0.33	3.00	3.00	1000	0.1 in group, 0.5 across groups
36	C	5	4	5	3	2	3	1.00	1.00	1.00	1000	0.3 in group, 0.7 across groups
37	C	5	4	5	3	2	3	0.67	1.50	1.50	1000	0.3 in group, 0.7 across groups
38	C	5	4	5	3	2	3	0.50	2.00	2.00	1000	0.3 in group, 0.7 across groups
39	C	5	4	5	3	2	3	0.40	2.50	2.50	1000	0.3 in group, 0.7 across groups
40	C	5	4	5	3	2	3	0.33	3.00	3.00	1000	0.3 in group, 0.7 across groups
41	C	5	4	5	3	2	3	1.00	1.00	1.00	1000	0.5 in group, 0.9 across groups
42	C	5	4	5	3	2	3	0.67	1.50	1.50	1000	0.5 in group, 0.9 across groups
43	C	5	4	5	3	2	3	0.50	2.00	2.00	1000	0.5 in group, 0.9 across groups
44	C	5	4	5	3	2	3	0.40	2.50	2.50	1000	0.5 in group, 0.9 across groups
45	C	5	4	5	3	2	3	0.33	3.00	3.00	1000	0.5 in group, 0.9 across groups
46	D	5	4	5	3	2	3	1.00	1.00	1.00	500	0.1 in group, 0.5 across groups
47	D	5	4	5	3	2	3	0.67	1.50	1.50	500	0.1 in group, 0.5 across groups
48	D	5	4	5	3	2	3	0.50	2.00	2.00	500	0.1 in group, 0.5 across groups
49	D	5	4	5	3	2	3	0.40	2.50	2.50	500	0.1 in group, 0.5 across groups
50	D	5	4	5	3	2	3	0.33	3.00	3.00	500	0.1 in group, 0.5 across groups
51	D	5	4	5	3	2	3	1.00	1.00	1.00	500	0.3 in group, 0.7 across groups
52	D	5	4	5	3	2	3	0.67	1.50	1.50	500	0.3 in group, 0.7 across groups
53	D	5	4	5	3	2	3	0.50	2.00	2.00	500	0.3 in group, 0.7 across groups
54	D	5	4	5	3	2	3	0.40	2.50	2.50	500	0.3 in group, 0.7 across groups
55	D	5	4	5	3	2	3	0.33	3.00	3.00	500	0.3 in group, 0.7 across groups
56	D	5	4	5	3	2	3	1.00	1.00	1.00	500	0.5 in group, 0.9 across groups
57	D	5	4	5	3	2	3	0.67	1.50	1.50	500	0.5 in group, 0.9 across groups
58	D	5	4	5	3	2	3	0.50	2.00	2.00	500	0.5 in group, 0.9 across groups
59	D	5	4	5	3	2	3	0.40	2.50	2.50	500	0.5 in group, 0.9 across groups
60	D	5	4	5	3	2	3	0.33	3.00	3.00	500	0.5 in group, 0.9 across groups

Table S2: List of chemicals and their group used in the CCLS analysis

Chemical	Chemical Group
PCB-118	PCB
PCB-138	PCB
PCB-153	PCB
PCB-180	PCB
DDE	Insecticide
DDT	Insecticide
Cyfluthrin(I)	Insecticide
Cyfluthrin(II)	Insecticide
Cyfluthrin(III)	Insecticide
Cyfluthrin(IV)	Insecticide
Carbaryl	Insecticide
Propoxur	Insecticide
Pentachlorophenol	Insecticide
gamma-Chlordane	Insecticide
alpha-Chlordane	Insecticide
Chlorpyrifos	Insecticide
Diazinon	Insecticide
Phosmet	Insecticide
cis-Permethrin	Insecticide
Methoxychlor	Insecticide
Cypermethrin(I)	Insecticide
Cypermethrin(II)	Insecticide
Cypermethrin(III)	Insecticide
Cypermethrin(IV)	Insecticide
trans-Permethrin	Insecticide
Piperonyl butoxide	Insecticide
o-Phenylphenol	Herbicide
Trifluralin	Herbicide
Simazine	Herbicide
mCPP	Herbicide
Dicamba	Herbicide
Dacthal	Herbicide
2,4-D	Herbicide
As	Metals
Cr	Metals
Cu	Metals
Pb	Metals
Sn	Metals
W	Metals
Zn	Metals

Indeno(1,2,3-c,d)pyrene	PAH
Dibenz(ah)anthracene	PAH
Dibenzo(ae)pyrene	PAH
Coronene	PAH
Benzo(a)anthracene	PAH
Benzo(a)pyrene	PAH
Benzo(b)fluoranthene	PAH
Nicotine	Tobacco
Cotinine	Tobacco

Table S3: Estimated odds ratio (OR) (averaged over 100 replicates) and power from the Bayesian group index model and group weighted quantile sum (GWQS) for Scenario A

**Parameter**	**Bayesian Group Index**		**GWQS**	
Weak Correlation	Estimated OR	Power	Estimated OR	Power
$\exp(\beta_1)$= 1.00<br>$\exp(\beta_2)$= 1.00	1.0057<br>1.0032	0.01<br>0.03	1.0194<br>1.0169	0.02<br>0.09
$\exp(\beta_1)$= 0.67<br>$\exp(\beta_2)$= 1.50	0.6546<br>1.5458	0.99<br>1.00	0.6654<br>1.5449	0.92<br>1.00
$\exp(\beta_1)$= 0.50<br>$\exp(\beta_2)$= 2.00	0.4794<br>2.0883	1.00<br>1.00	0.4972<br>2.0218	1.00<br>1.00
$\exp(\beta_1)$= 0.40<br>$\exp(\beta_2)$= 2.50	0.3795<br>2.6347	1.00<br>1.00	0.3996<br>2.5182	1.00<br>1.00
$\exp(\beta_1)$= 0.33<br>$\exp(\beta_2)$= 3.00	0.3148<br>3.1550	1.00<br>1.00	0.3326<br>3.0067	1.00<br>1.00
Moderate Correlation	Estimated OR	Power	Estimated OR	Power
$\exp(\beta_1)$= 1.00<br>$\exp(\beta_2)$= 1.00	1.0086<br>1.0026	0.03<br>0.02	1.0107<br>0.9981	0.06<br>0.07
$\exp(\beta_1)$= 0.67<br>$\exp(\beta_2)$= 1.50	0.6664<br>1.5158	1.00<br>1.00	0.6799<br>1.4668	0.98<br>0.97
$\exp(\beta_1)$= 0.50<br>$\exp(\beta_2)$= 2.00	0.4965<br>2.0446	1.00<br>1.00	0.5160<br>1.9785	1.00<br>1.00
$\exp(\beta_1)$= 0.40<br>$\exp(\beta_2)$= 2.50	0.3925<br>2.5963	1.00<br>1.00	0.4113<br>2.4797	1.00<br>1.00
$\exp(\beta_1)$= 0.33<br>$\exp(\beta_2)$= 3.00	0.3189<br>3.1939	1.00<br>1.00	0.3356<br>3.0480	1.00<br>1.00
Strong Correlation	Estimated OR	Power	Estimated OR	Power
$\exp(\beta_1)$= 1.00<br>$\exp(\beta_2)$= 1.00	1.0096<br>0.9967	0.07<br>0.04	1.0171<br>0.9949	0.04<br>0.06
$\exp(\beta_1)$= 0.67<br>$\exp(\beta_2)$= 1.50	0.6659<br>1.5154	1.00<br>1.00	0.6755<br>1.4848	0.99<br>1.00
$\exp(\beta_1)$= 0.50<br>$\exp(\beta_2)$= 2.00	0.5024<br>1.9955	1.00<br>1.00	0.5101<br>1.9624	1.00<br>1.00
$\exp(\beta_1)$= 0.40<br>$\exp(\beta_2)$= 2.50	0.4002<br>2.5067	1.00<br>1.00	0.4173<br>2.4172	1.00<br>1.00
$\exp(\beta_1)$= 0.33<br>$\exp(\beta_2)$= 3.00	0.3278<br>3.0642	1.00<br>1.00	0.3389<br>2.9603	1.00<br>1.00

Table S4: Estimated odds ratio (OR) (averaged over 100 replicates) and power from the Bayesian group index model and group weighted quantile sum (GWQS) for Scenario B

**Parameter**	**Bayesian Group Index**		**GWQS**	
Weak Correlation	Estimated OR	Power	Estimated OR	Power
$\exp(\beta_1)$= 1.00<br>$\exp(\beta_2)$= 1.00<br>$\exp(\beta_3)$= 1.00	0.9957<br>0.9989<br>1.0087	0.05<br>0.05<br>0.04	1.0106<br>0.9855<br>1.0169	0.07<br>0.04<br>0.04
$\exp(\beta_1)$= 0.67<br>$\exp(\beta_2)$= 1.50<br>$\exp(\beta_3)$= 1.50	0.6813<br>1.5179<br>1.5018	0.99<br>1.00<br>1.00	0.7133<br>1.4876<br>1.4580	0.81<br>0.94<br>0.89
$\exp(\beta_1)$= 0.50<br>$\exp(\beta_2)$= 2.00<br>$\exp(\beta_3)$= 2.00	0.4970<br>2.0648<br>2.0358	1.00<br>1.00<br>1.00	0.5278<br>2.0169<br>1.9995	0.99<br>1.00<br>1.00
$\exp(\beta_1)$= 0.40<br>$\exp(\beta_2)$= 2.50<br>$\exp(\beta_3)$= 2.50	0.3896<br>2.6110<br>2.5708	1.00<br>1.00<br>1.00	0.4212<br>2.5161<br>2.4726	1.00<br>1.00<br>1.00
$\exp(\beta_1)$= 0.33<br>$\exp(\beta_2)$= 3.00<br>$\exp(\beta_3)$= 3.00	0.3274<br>3.1119<br>3.0886	1.00<br>1.00<br>1.00	0.3518<br>3.0181<br>2.8874	1.00<br>1.00<br>1.00
Moderate Correlation	Estimated OR	Power	Estimated OR	Power
$\exp(\beta_1)$= 1.00<br>$\exp(\beta_2)$= 1.00<br>$\exp(\beta_3)$= 1.00	0.9938<br>1.0001<br>1.0038	0.04<br>0.01<br>0.04	1.0048<br>1.0031<br>1.0133	0.04<br>0.04<br>0.04
$\exp(\beta_1)$= 0.67<br>$\exp(\beta_2)$= 1.50<br>$\exp(\beta_3)$= 1.50	0.6760<br>1.5068<br>1.4938	1.00<br>1.00<br>1.00	0.7035<br>1.4700<br>1.4794	0.91<br>0.98<br>0.96
$\exp(\beta_1)$= 0.50<br>$\exp(\beta_2)$= 2.00<br>$\exp(\beta_3)$= 2.00	0.5063<br>1.9934<br>2.0129	1.00<br>1.00<br>1.00	0.5346<br>1.9282<br>1.9933	1.00<br>1.00<br>1.00
$\exp(\beta_1)$= 0.40<br>$\exp(\beta_2)$= 2.50<br>$\exp(\beta_3)$= 2.50	0.4053<br>2.5456<br>2.5307	1.00<br>1.00<br>1.00	0.4260<br>2.4817<br>2.4693	1.00<br>1.00<br>1.00
$\exp(\beta_1)$= 0.33<br>$\exp(\beta_2)$= 3.00<br>$\exp(\beta_3)$= 3.00	0.3359<br>3.0217<br>3.0009	1.00<br>1.00<br>1.00	0.3609<br>2.9188<br>2.8864	1.00<br>1.00<br>1.00
Strong Correlation	Estimated OR	Power	Estimated OR	Power
$\exp(\beta_1)$= 1.00<br>$\exp(\beta_2)$= 1.00<br>$\exp(\beta_3)$= 1.00	0.9968<br>1.0047<br>1.0011	0.07<br>0.03<br>0.03	1.0071<br>1.0007<br>1.0030	0.09<br>0.04<br>0.04
$\exp(\beta_1)$= 0.67<br>$\exp(\beta_2)$= 1.50<br>$\exp(\beta_3)$= 1.50	0.6716<br>1.4989<br>1.5049	1.00<br>1.00<br>1.00	0.6891<br>1.4658<br>1.4920	0.91<br>0.96<br>0.95
$\exp(\beta_1)$= 0.50<br>$\exp(\beta_2)$= 2.00<br>$\exp(\beta_3)$= 2.00	0.5046<br>2.0131<br>1.9934	1.00<br>1.00<br>1.00	0.5295<br>1.9769<br>1.9303	1.00<br>1.00<br>1.00
$\exp(\beta_1)$= 0.40	0.4041	1.00	0.4278	1.00

exp(β_2)= 2.50<br>exp(β_3)= 2.50	2.5071<br>2.4974	1.00<br>1.00	2.4496<br>2.4257	1.00<br>1.00
exp(β_1)= 0.33<br>exp(β_2)= 3.00<br>exp(β_3)= 3.00	0.3392<br>3.0142<br>2.9794	1.00<br>1.00<br>1.00	0.3634<br>2.9282<br>2.8888	1.00<br>1.00<br>1.00

Table S5: Estimated odds ratio (OR) and power values for the Bayesian group index model and group weighted quantile sum (GWQS) regression for Scenario C

**Parameter**	**Bayesian Group Index**		**GWQS**	
Weak Correlation	Estimated OR	Power	Estimated OR	Power
exp(β_1)= 1.00<br>exp(β_2)= 1.00<br>exp(β_3)= 1.00	0.9957<br>0.9989<br>1.0087	0.05<br>0.05<br>0.04	1.0106<br>0.9855<br>1.0169	0.07<br>0.04<br>0.04
exp(β_1)= 0.67<br>exp(β_2)= 1.50<br>exp(β_3)= 1.50	0.6189<br>1.5803<br>1.6205	1.00<br>1.00<br>1.00	0.6286<br>1.5599<br>1.6251	0.98<br>0.96<br>1.00
exp(β_1)= 0.50<br>exp(β_2)= 2.00<br>exp(β_3)= 2.00	0.4521<br>2.1360<br>2.1724	1.00<br>1.00<br>1.00	0.4609<br>2.1173<br>2.1751	1.00<br>1.00<br>1.00
exp(β_1)= 0.40<br>exp(β_2)= 2.50<br>exp(β_3)= 2.50	0.3599<br>2.6983<br>2.7867	1.00<br>1.00<br>1.00	0.3712<br>2.6758<br>2.7812	1.00<br>1.00<br>1.00
exp(β_1)= 0.33<br>exp(β_2)= 3.00<br>exp(β_3)= 3.00	0.2950<br>3.2694<br>3.3399	1.00<br>1.00<br>1.00	0.3110<br>3.1715<br>3.3223	1.00<br>1.00<br>1.00
Moderate Correlation	Estimated OR	Power	Estimated OR	Power
exp(β_1)= 1.00<br>exp(β_2)= 1.00<br>exp(β_3)= 1.00	0.9938<br>1.0001<br>1.0038	0.04<br>0.01<br>0.04	1.0048<br>1.0031<br>1.0133	0.04<br>0.04<br>0.04
exp(β_1)= 0.67<br>exp(β_2)= 1.50<br>exp(β_3)= 1.50	0.6388<br>1.5411<br>1.5644	1.00<br>1.00<br>1.00	0.6536<br>1.5267<br>1.5383	1.00<br>0.99<br>1.00
exp(β_1)= 0.50<br>exp(β_2)= 2.00<br>exp(β_3)= 2.00	0.4681<br>2.0944<br>2.1067	1.00<br>1.00<br>1.00	0.4797<br>2.0665<br>2.0759	1.00<br>1.00<br>1.00
exp(β_1)= 0.40<br>exp(β_2)= 2.50<br>exp(β_3)= 2.50	0.3757<br>2.6020<br>2.6310	1.00<br>1.00<br>1.00	0.3928<br>2.5400<br>2.5671	1.00<br>1.00<br>1.00
exp(β_1)= 0.33<br>exp(β_2)= 3.00<br>exp(β_3)= 3.00	0.3077<br>3.2028<br>3.2282	1.00<br>1.00<br>1.00	0.3216<br>3.0951<br>3.1515	1.00<br>1.00<br>1.00
Strong Correlation	Estimated OR	Power	Estimated OR	Power
exp(β_1)= 1.00<br>exp(β_2)= 1.00<br>exp(β_3)= 1.00	0.9968<br>1.0047<br>1.0011	0.07<br>0.03<br>0.03	0.9985<br>1.0092<br>0.9992	0.03<br>0.06<br>0.05

$\exp(\beta_1)$= 0.67<br>$\exp(\beta_2)$= 1.50<br>$\exp(\beta_3)$= 1.50	0.6525<br>1.5276<br>1.5277	1.00<br>1.00<br>1.00	0.6736<br>1.5026<br>1.5119	0.95<br>0.96<br>0.98
$\exp(\beta_1)$= 0.50<br>$\exp(\beta_2)$= 2.00<br>$\exp(\beta_3)$= 2.00	0.4833<br>2.0311<br>2.0567	1.00<br>1.00<br>1.00	0.4966<br>2.0098<br>2.0219	1.00<br>1.00<br>1.00
$\exp(\beta_1)$= 0.40<br>$\exp(\beta_2)$= 2.50<br>$\exp(\beta_3)$= 2.50	0.3836<br>2.5435<br>2.5808	1.00<br>1.00<br>1.00	0.4066<br>2.4508<br>2.5250	1.00<br>1.00<br>1.00
$\exp(\beta_1)$= 0.33<br>$\exp(\beta_2)$= 3.00<br>$\exp(\beta_3)$= 3.00	0.3188<br>3.0729<br>3.0978	1.00<br>1.00<br>1.00	0.3475<br>2.9347<br>2.9785	1.00<br>1.00<br>1.00

Table S6: MSE and for the Bayesian group index model and group weighted quantile sum (GWQS) for Scenario A

**Parameter**	**Bayesian Group Index**		**GWQS**	
Weak Correlation	MSE	Bias	MSE	Bias
$\exp(\beta_1)$= 1.00<br>$\exp(\beta_2)$= 1.00	0.0049<br>0.0055	0.0032<br>0.0004	0.0122<br>0.0185	0.0132<br>0.0075
$\exp(\beta_1)$= 0.67<br>$\exp(\beta_2)$= 1.50	0.0094<br>0.0075	-0.0229<br>0.0267	0.0164<br>0.0131	-0.0101<br>0.0228
$\exp(\beta_1)$= 0.50<br>$\exp(\beta_2)$= 2.00	0.0098<br>0.0092	-0.0460<br>0.0394	0.0205<br>0.0157	-0.0155<br>0.0029
$\exp(\beta_1)$= 0.40<br>$\exp(\beta_2)$= 2.50	0.0121<br>0.0108	-0.0570<br>0.0482	0.0220<br>0.0202	-0.0118<br>-0.0030
$\exp(\beta_1)$= 0.33<br>$\exp(\beta_2)$= 3.00	0.0169<br>0.0115	-0.0636<br>0.0456	0.0239<br>0.0198	-0.0140<br>-0.0078
Moderate Correlation	MSE	Bias	MSE	Bias
$\exp(\beta_1)$= 1.00<br>$\exp(\beta_2)$= 1.00	0.0046<br>0.0050	0.0062<br>0.0001	0.0101<br>0.0117	0.0055<br>-0.0077
$\exp(\beta_1)$= 0.67<br>$\exp(\beta_2)$= 1.50	0.0055<br>0.0058	-0.0031<br>0.0076	0.0114<br>0.0117	0.0140<br>-0.0278
$\exp(\beta_1)$= 0.50<br>$\exp(\beta_2)$= 2.00	0.0080<br>0.0074	-0.0109<br>0.0186	0.0161<br>0.0128	0.0238<br>-0.0171
$\exp(\beta_1)$= 0.40<br>$\exp(\beta_2)$= 2.50	0.0094<br>0.0097	-0.0235<br>0.0335	0.0153<br>0.0163	0.0206<br>-0.0162
$\exp(\beta_1)$= 0.33<br>$\exp(\beta_2)$= 3.00	0.0113<br>0.0127	-0.0486<br>0.0580	0.0192<br>0.0199	-0.0027<br>0.0060
Strong Correlation	MSE	Bias	MSE	Bias
$\exp(\beta_1)$= 1.00<br>$\exp(\beta_2)$= 1.00	0.0047<br>0.0040	0.0072<br>-0.0053	0.0101<br>0.0097	0.0119<br>-0.0099
$\exp(\beta_1)$= 0.67<br>$\exp(\beta_2)$= 1.50	0.0062<br>0.0055	-0.0043<br>0.0075	0.0100<br>0.0077	0.0082<br>-0.0140
$\exp(\beta_1)$= 0.50<br>$\exp(\beta_2)$= 2.00	0.0064<br>0.0065	0.0015<br>-0.0055	0.0135<br>0.0145	0.0132<br>-0.0259
$\exp(\beta_1)$= 0.40<br>$\exp(\beta_2)$= 2.50	0.0076<br>0.0071	-0.0033<br>-0.0009	0.0179<br>0.0158	0.0340<br>-0.0408
$\exp(\beta_1)$= 0.33<br>$\exp(\beta_2)$= 3.00	0.0087<br>0.0103	-0.0208<br>0.0161	0.0176<br>0.0202	0.0077<br>-0.0233

Table S7: MSE and Bias for the Bayesian group index model and group weighted quantile sum (GWQS) for Scenario B

**Parameter**	**Bayesian Group Index**		**GWQS**	
Weak Correlation	MSE	Bias	MSE	Bias
$\exp(\beta_1)$= 1.00	0.0075	-0.0081	0.0162	0.0024
$\exp(\beta_2)$= 1.00	0.0056	-0.0039	0.0131	-0.0210
$\exp(\beta_3)$= 1.00	0.0074	0.0049	0.0151	0.0093
$\exp(\beta_1)$= 0.67	0.0072	0.0183	0.0173	0.0608
$\exp(\beta_2)$= 1.50	0.0079	0.0079	0.0138	-0.0151
$\exp(\beta_3)$= 1.50	0.0077	-0.0026	0.0138	-0.0347
$\exp(\beta_1)$= 0.50	0.0106	-0.0113	0.0238	0.0431
$\exp(\beta_2)$= 2.00	0.0113	0.0266	0.0160	0.0002
$\exp(\beta_3)$= 2.00	0.0107	0.0125	0.0169	-0.0088
$\exp(\beta_1)$= 0.40	0.0120	-0.0319	0.0217	0.0418
$\exp(\beta_2)$= 2.50	0.0098	0.0393	0.0205	-0.0038
$\exp(\beta_3)$= 2.50	0.0129	0.0216	0.0228	-0.0223
$\exp(\beta_1)$= 0.33	0.0148	-0.0249	0.0243	0.0425
$\exp(\beta_2)$= 3.00	0.0118	0.0312	0.0276	-0.0078
$\exp(\beta_3)$= 3.00	0.0133	0.0227	0.0290	-0.0514
Moderate Correlation	MSE	Bias	MSE	Bias
$\exp(\beta_1)$= 1.00	0.0049	-0.0086	0.0115	-0.0010
$\exp(\beta_2)$= 1.00	0.0040	-0.0020	0.0101	-0.0019
$\exp(\beta_3)$= 1.00	0.0045	0.0015	0.0101	0.0081
$\exp(\beta_1)$= 0.67	0.0058	0.0111	0.0148	0.0475
$\exp(\beta_2)$= 1.50	0.0064	0.0013	0.0124	-0.0263
$\exp(\beta_3)$= 1.50	0.0050	-0.0066	0.0112	-0.0193
$\exp(\beta_1)$= 0.50	0.0073	0.0090	0.0191	0.0588
$\exp(\beta_2)$= 2.00	0.0068	-0.0067	0.0121	-0.0417
$\exp(\beta_3)$= 2.00	0.0070	0.0029	0.0146	-0.0105
$\exp(\beta_1)$= 0.40	0.0111	0.0077	0.0246	0.0522
$\exp(\beta_2)$= 2.50	0.0077	0.0143	0.0129	-0.0137
$\exp(\beta_3)$= 2.50	0.0112	0.0066	0.0236	-0.0240
$\exp(\beta_1)$= 0.33	0.0107	0.0025	0.0320	0.0660
$\exp(\beta_2)$= 3.00	0.0098	0.0023	0.0207	-0.0374
$\exp(\beta_3)$= 3.00	0.0109	-0.0053	0.0239	-0.0496
Strong Correlation	MSE	Bias	MSE	Bias
$\exp(\beta_1)$= 1.00	0.0062	-0.0063	0.0148	-0.0004
$\exp(\beta_2)$= 1.00	0.0040	0.0026	0.0098	-0.0041
$\exp(\beta_3)$= 1.00	0.0047	-0.0013	0.0098	-0.0020
$\exp(\beta_1)$= 0.67	0.0062	0.0043	0.0144	0.0262
$\exp(\beta_2)$= 1.50	0.0056	-0.0035	0.0112	-0.0282
$\exp(\beta_3)$= 1.50	0.0058	0.0004	0.0115	-0.0110
$\exp(\beta_1)$= 0.50	0.0065	0.0060	0.0156	0.0510
$\exp(\beta_2)$= 2.00	0.0070	0.0030	0.0149	-0.0189
$\exp(\beta_3)$= 2.00	0.0061	-0.0063	0.0130	-0.0411
$\exp(\beta_1)$= 0.40	0.0090	0.0057	0.0207	0.0585

$\exp(\beta_2)$= 2.50<br>$\exp(\beta_3)$= 2.50	0.0082<br>0.0072	-0.0013<br>-0.0047	0.0156<br>0.0157	-0.0278<br>-0.0374
$\exp(\beta_1)$= 0.33<br>$\exp(\beta_2)$= 3.00<br>$\exp(\beta_3)$= 3.00	0.0103<br>0.0100<br>0.0076	0.0123<br>-0.0003<br>-0.0107	0.0259<br>0.0201<br>0.0159	0.0765<br>-0.0337<br>-0.0448

Table S8: MSE and bias for effect estimates from the Bayesian group index model and group weighted quantile sum (GWQS) regression for Scenario C

**Parameter**	**Bayesian Group Index**		**GWQS**	
Weak Correlation	MSE	Bias	MSE	Bias
$\exp(\beta_1)$= 1.00<br>$\exp(\beta_2)$= 1.00<br>$\exp(\beta_3)$= 1.00	0.0075<br>0.0056<br>0.0074	-0.0081<br>-0.0039<br>0.0049	0.0162<br>0.0131<br>0.0151	0.0024<br>-0.0210<br>0.0093
$\exp(\beta_1)$= 0.67<br>$\exp(\beta_2)$= 1.50<br>$\exp(\beta_3)$= 1.50	0.0110<br>0.0078<br>0.0106	-0.0769<br>0.0494<br>0.0748	0.0183<br>0.0145<br>0.0182	-0.0658<br>0.0324<br>0.0736
$\exp(\beta_1)$= 0.50<br>$\exp(\beta_2)$= 2.00<br>$\exp(\beta_3)$= 2.00	0.0176<br>0.0093<br>0.0118	-0.1041<br>0.0631<br>0.0800	0.0233<br>0.0141<br>0.0180	-0.0891<br>0.0512<br>0.0779
$\exp(\beta_1)$= 0.40<br>$\exp(\beta_2)$= 2.50<br>$\exp(\beta_3)$= 2.50	0.0202<br>0.0122<br>0.0191	-0.1097<br>0.0728<br>0.1044	0.0275<br>0.0234<br>0.0252	-0.0845<br>0.0577<br>0.0988
$\exp(\beta_1)$= 0.33<br>$\exp(\beta_2)$= 3.00<br>$\exp(\beta_3)$= 3.00	0.0225<br>0.0152<br>0.0200	-0.1255<br>0.0817<br>0.1025	0.0241<br>0.0219<br>0.0237	-0.0781<br>0.0455<br>0.0946
Moderate Correlation	MSE	Bias	MSE	Bias
$\exp(\beta_1)$= 1.00<br>$\exp(\beta_2)$= 1.00<br>$\exp(\beta_3)$= 1.00	0.0049<br>0.0040<br>0.0045	-0.0086<br>-0.0020<br>0.0015	0.0115<br>0.0101<br>0.0101	-0.0010<br>-0.0019<br>0.0081
$\exp(\beta_1)$= 0.67<br>$\exp(\beta_2)$= 1.50<br>$\exp(\beta_3)$= 1.50	0.0069<br>0.0054<br>0.0062	-0.0451<br>0.0246<br>0.0397	0.0111<br>0.0112<br>0.0089	-0.0250<br>0.0121<br>0.0209
$\exp(\beta_1)$= 0.50<br>$\exp(\beta_2)$= 2.00<br>$\exp(\beta_3)$= 2.00	0.0104<br>0.0088<br>0.0073	-0.0688<br>0.0427<br>0.0495	0.0149<br>0.0133<br>0.0137	-0.0478<br>0.0264<br>0.0309
$\exp(\beta_1)$= 0.40<br>$\exp(\beta_2)$= 2.50<br>$\exp(\beta_3)$= 2.50	0.0134<br>0.0078<br>0.0092	-0.0670<br>0.0367<br>0.0476	0.0211<br>0.0139<br>0.0147	-0.0285<br>0.0089<br>0.0193
$\exp(\beta_1)$= 0.33<br>$\exp(\beta_2)$= 3.00<br>$\exp(\beta_3)$= 3.00	0.0209<br>0.0127<br>0.0132	-0.0868<br>0.0609<br>0.0690	0.0267<br>0.0167<br>0.0187	-0.0480<br>0.0230<br>0.0407
Strong Correlation	MSE	Bias	MSE	Bias
$\exp(\beta_1)$= 1.00	0.0062	-0.0063	0.0106	-0.0067

$\exp(\beta_2)$= 1.00<br>$\exp(\beta_3)$= 1.00	0.0040<br>0.0047	0.0026<br>-0.0013	0.0109<br>0.0111	0.0037<br>-0.0063
$\exp(\beta_1)$= 0.67<br>$\exp(\beta_2)$= 1.50<br>$\exp(\beta_3)$= 1.50	0.0051<br>0.0078<br>0.0055	-0.0237<br>0.0145<br>0.0157	0.0085<br>0.0128<br>0.0095	0.0061<br>-0.0047<br>0.0032
$\exp(\beta_1)$= 0.50<br>$\exp(\beta_2)$= 2.00<br>$\exp(\beta_3)$= 2.00	0.0079<br>0.0079<br>0.0085	-0.0373<br>0.0115<br>0.0239	0.0135<br>0.0151<br>0.0152	-0.0134<br>-0.0026<br>0.0031
$\exp(\beta_1)$= 0.40<br>$\exp(\beta_2)$= 2.50<br>$\exp(\beta_3)$= 2.50	0.0106<br>0.0080<br>0.0083	-0.0462<br>0.0134<br>0.0280	0.0207<br>0.0170<br>0.0139	0.0060<br>-0.0279<br>0.0029
$\exp(\beta_1)$= 0.33<br>$\exp(\beta_2)$= 3.00<br>$\exp(\beta_3)$= 3.00	0.0132<br>0.0100<br>0.0103	-0.0500<br>0.0192<br>0.0272	0.0243<br>0.0217<br>0.0185	0.0300<br>-0.0323<br>-0.0165

Table S9: Sensitivity and specificity for the Bayesian group index model and group weighted quantile sum (GWQS) regression for simulation scenarios A-C

**Effect Size**		**Bayesian Group Index**		**GWQS**	
Scenario A	Correlation	Sensitivity	Specificity	Sensitivity	Specificity
OR = 1.00	Weak<br>Moderate<br>Strong	0.400<br>0.413<br>0.430	0.606<br>0.562<br>0.532	0.385<br>0.408<br>0.390	0.602<br>0.606<br>0.624
OR = 1.50	Weak<br>Moderate<br>Strong	0.893<br>0.768<br>0.673	0.968<br>0.944<br>0.858	0.848<br>0.708<br>0.608	0.898<br>0.846<br>0.748
OR = 2.00	Weak<br>Moderate<br>Strong	0.985<br>0.908<br>0.788	0.992<br>0.980<br>0.936	0.960<br>0.850<br>0.748	0.964<br>0.902<br>0.802
OR = 2.50	Weak<br>Moderate<br>Strong	1.000<br>0.968<br>0.860	1.000<br>0.988<br>0.968	0.993<br>0.930<br>0.813	0.998<br>0.960<br>0.896
OR = 3.00	Weak<br>Moderate<br>Strong	1.000<br>0.990<br>0.933	0.998<br>1.000<br>0.984	0.995<br>0.963<br>0.860	0.998<br>0.978<br>0.908
Scenario B	Correlation	Sensitivity	Specificity	Sensitivity	Specificity
OR = 1.00	Weak<br>Moderate<br>Strong	0.376<br>0.424<br>0.413	0.615<br>0.592<br>0.565	0.401<br>0.416<br>0.386	0.635<br>0.632<br>0.613
OR = 1.50	Weak<br>Moderate<br>Strong	0.713<br>0.625<br>0.553	0.953<br>0.910<br>0.835	0.688<br>0.605<br>0.535	0.883<br>0.825<br>0.752
OR = 2.00	Weak<br>Moderate<br>Strong	0.858<br>0.773<br>0.651	0.985<br>0.967<br>0.897	0.810<br>0.740<br>0.613	0.947<br>0.918<br>0.820

OR = 2.50	Weak<br>Moderate<br>Strong	0.919<br>0.819<br>0.710	1.000<br>0.980<br>0.950	0.873<br>0.774<br>0.665	0.987<br>0.953<br>0.880
OR = 3.00	Weak<br>Moderate<br>Strong	0.969<br>0.849<br>0.735	1.000<br>0.988<br>0.962	0.908<br>0.821<br>0.706	0.988<br>0.965<br>0.892
Scenario C	Correlation	Sensitivity	Specificity	Sensitivity	Specificity
OR = 1.00	Weak<br>Moderate<br>Strong	0.393<br>0.443<br>0.440	0.624<br>0.590<br>0.583	0.443<br>0.433<br>0.410	0.630<br>0.615<br>0.598
OR = 1.50	Weak<br>Moderate<br>Strong	1.000<br>0.993<br>0.977	0.994<br>0.981<br>0.915	1.000<br>0.993<br>0.923	0.935<br>0.874<br>0.810
OR = 2.00	Weak<br>Moderate<br>Strong	1.000<br>1.000<br>1.000	0.996<br>0.996<br>0.978	1.000<br>1.000<br>0.980	0.980<br>0.962<br>0.888
OR = 2.50	Weak<br>Moderate<br>Strong	1.000<br>1.000<br>1.000	0.999<br>0.998<br>0.993	1.000<br>1.000<br>1.000	0.992<br>0.977<br>0.926
OR = 3.00	Weak<br>Moderate<br>Strong	1.000<br>1.000<br>1.000	0.998<br>1.000<br>0.996	1.000<br>1.000<br>1.000	0.995<br>0.985<br>0.937

Table S10: Odds ratio estimates for chemical groups and demographic covariates from the Bayesian group index model for subjects with different residence since birth

**Variable**	**Odds Ratio**	**2.5% CI**	**97.5% CI**
PCBs	1.02	0.72	1.46
Insecticides	0.65	0.32	1.22
Herbicides	0.89	0.55	1.45
Metals	0.74	0.50	1.12
PAHs	1.25	0.91	1.75
Tobacco	0.90	0.68	1.15
Child's age	1.11	0.97	1.30
Female	0.97	0.59	1.58
Child's Ethnicity			
Hispanic vs White Non-Hispanic	1.19	0.65	2.30
Other Non-Hispanic vs White Non-Hispanic	1.12	0.59	2.17
Household Income			
$15,000 - $29,999 vs Less than $15,000	0.71	0.24	1.89
$30,000 - $44,999 vs Less than $15,000	0.92	0.31	2.53
$45,000 - $59,999 vs Less than $15,000	0.67	0.21	1.92
$60,000 - $74,999 vs Less than $15,000	**0.25**	**0.06**	**0.90**
$75,000 or more vs Less than $15,000	0.42	0.14	1.15
Mother's education			
High school vs Less than high school	0.73	0.20	2.09
Some college vs Less than high school	0.66	0.18	1.96
Bachelor's or higher vs Less than high school	0.93	0.24	2.97
Mother's age	1.00	0.96	1.05

Figure S1: Forest plot of chemical group effects for childhood leukemia in children who changed residence since birth

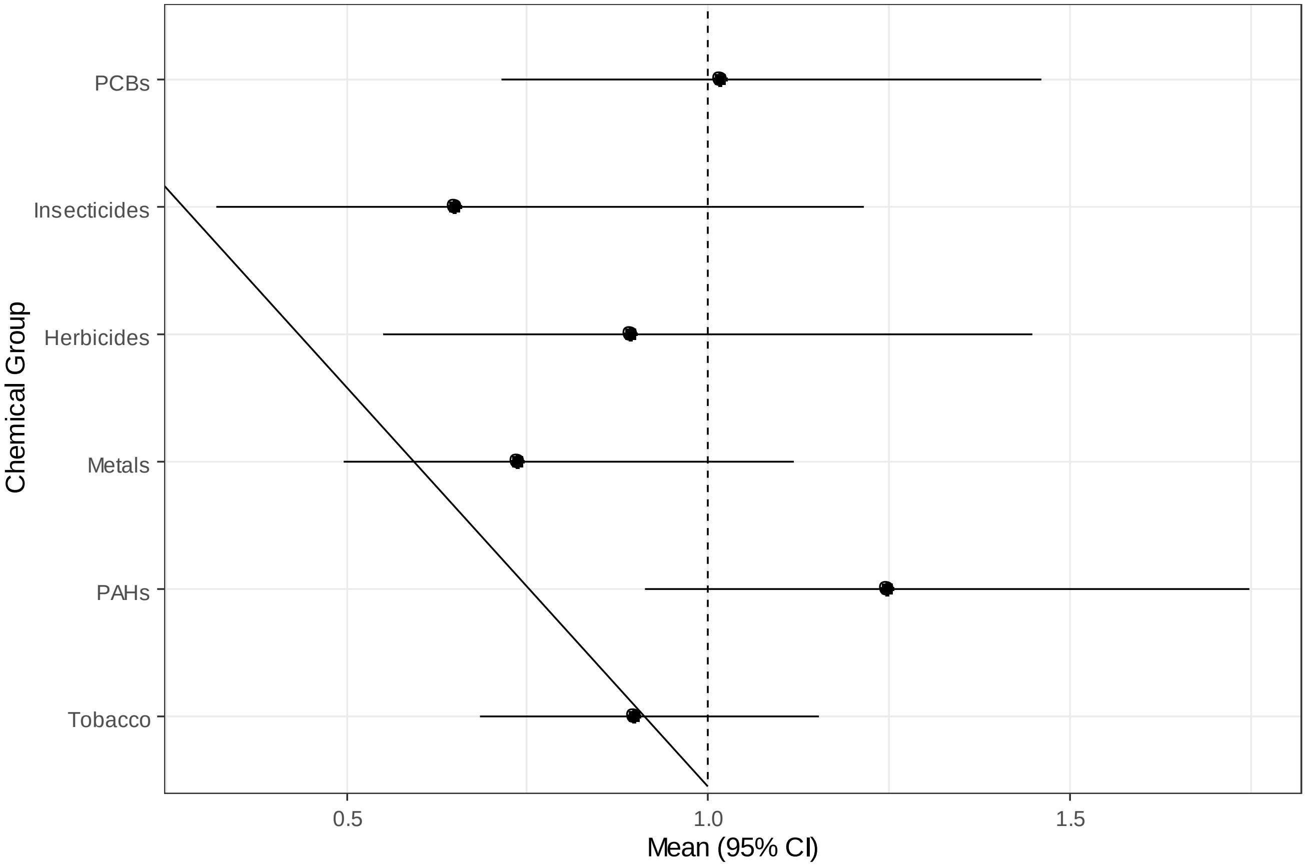

Figure S2: Estimated chemical weights for chemical groups from the Bayesian group index model for childhood leukemia in the CCLS in children who changed residence since birth

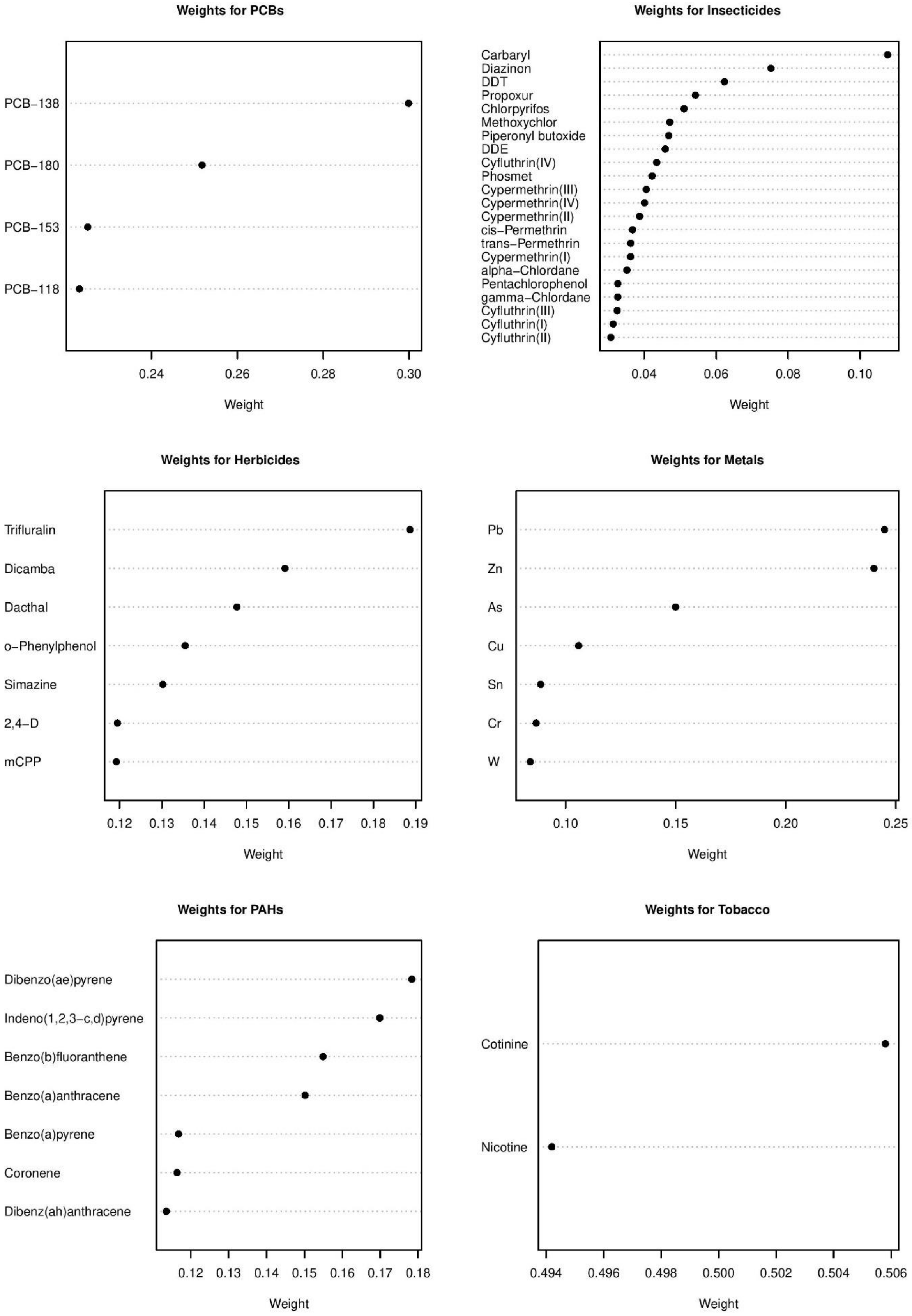

Weights for PCBs
PCB−138
PCB−180
PCB−153
PCB−118
0.24
0.26
0.28
0.30
Weight
Weights for Insecticides
Carbaryl
Diazinon
DDT
Propoxur
Chlorpyrifos
Methoxychlor
Piperonyl butoxide
DDE
Cyfluthrin(IV)
Phosmet
Cypermethrin(III)
Cypermethrin(IV)
Cypermethrin(II)
cis−Permethrin
trans−Permethrin
Cypermethrin(I)
alpha−Chlordane
Pentachlorophenol
gamma−Chlordane
Cyfluthrin(III)
Cyfluthrin(I)
Cyfluthrin(II)
0.04
0.06
0.08
0.10
Weight
Weights for Herbicides
Trifluralin
Dicamba
Dacthal
o−Phenylphenol
Simazine
2,4−D
mCPP
0.12
0.13
0.14
0.15
0.16
0.17
0.18
0.19
Weight
Weights for Metals
Pb
Zn
As
Cu
Sn
Cr
W
0.10
0.15
0.20
0.25
Weight
Weights for PAHs
Dibenzo(ae)pyrene
Indeno(1,2,3−c,d)pyrene
Benzo(b)fluoranthene
Benzo(a)anthracene
Benzo(a)pyrene
Coronene
Dibenz(ah)anthracene
0.12
0.13
0.14
0.15
0.16
0.17
0.18
Weight
Weights for Tobacco
Cotinine
Nicotine
0.494
0.496
0.498
0.500
0.502
0.504
0.506
Weight
